# The evaluation of vancomycin-resistant enterococci and carbapenamase producing Klebsiella colonization among ICU-Hospitalized Patients

**DOI:** 10.4314/ahs.v21i4.20

**Published:** 2021-12

**Authors:** Gökhan Karaşin, Yasemin Bayram, Mehmet Parlak, Cenk Aypak, Mustafa Akgül, Hüseyin Güdücüoğlu

**Affiliations:** 1 Microbiology Laboratory. Van Training and Research Hospital, 65300, Van, Turkey; 2 Department of Microbiology, Van Yüzüncü Yıl University, Medical Faculty, 65080, Van, Turkey; 3 Department of Family Medicine, University of Health Sciences, Ankara Dışkapı Yıldırım Beyazıt Training and Research Hospital, 06110, Ankara, Turkey

**Keywords:** Carbapenam resistant klebsiella pneumoniae, vancomycin-resistant enterococci, intensive care units

## Abstract

**Background:**

Multi-drug resistant organisms, especially Vancomycin-Resistant Enterococcus (VRE) and Carbapenam Resistant Klebsiella pneumoniae (KPC), are serious health threat. Early detection of resistant bacteria colonization among patients in intensive care units (ICUs) not only enables effective treatment but more importantly prevents disease and limits transmission. Therefore, we aimed to to assess the frequency of VRE and KPC colonization via rectal swab sampling.

**Methods:**

The study was carried out in ICUs of a tertiary hospital. Two rectal swab samples were collected within the first 24 hours of admission and another one was taken every subsequent 15 days to test for for VRE and KPC carriage.

**Results:**

A total 316 rectal swab samples taken from 230 patients. Forty-seven patients were screened at least 2 times. 183 patients were not further screened due to discharge, exitus or transfer to other wards. Thirty-six patients (16%) were determined to be VRE (+). The most frequently isolated strain was E. faecium (80.5%) and its most common genotype was VanA (87.5%). Seven patients (3%) were identified as KPC (+). OXA-48 type crbapenamase was confirmed in all KPC isolates.

**Conclusion:**

This study shows that VRE and KPC colonization continues to be a serious threat in ICUs.

## Introduction

In recent years, multi-drug resistant organisms (MDROs), especially Vancomycin-Resistant Enterococcus (VRE) and Carbapem resistant Klebsiella pneumoniae (KPC), are becoming more prevalent worldwide which leads to a serious public health threat^1^.

These bacteria are important opportunistic pathogens that have developed plasmid encoded resistance to numerous antibiotics. Therefore the treatment of infections caused by their resistant strains has become quite challenging, especially in tough settings such as intensive care units (ICU)^2^.

MDROs represent a substantial proportion of nosocomial infections in the ICU, including 10% to 16% of United States (US) device-related infections^3^. To prevent MDRO infections, effective and comprehensive precautions must be taken including antibiotic stewardship, infection control measures to prevent cross-transmission, and the implementation of rapid and accurate MDROs detection methods in the laboratory^4^. Patients who are colonized with MDROs serve inadvertently as potential reservoirs for transmission. Colonization pressure, or the proportion of patients in a given unit who are colonized with resistant bacteria, is an independent risk factor for transmission^5^. Therefore early detection of bacterial colonization in high-risk groups such as ICU patients has been proven to help prevent disease and dissemination^6^.

However, there have been relatively few reports on molecular characterization and clonal epidemiology of VRE and KPC colonization in our country. The purpose of this study was to assess the frequency of VRE and KPC colonization via rectal swab sampling among ICU patients. Samples with positive results were further analyzed for bacterial subtypes and resistance genes.

## Methods

### Study Settings

This study was conducted at ICU (cardiology, neurology, internal medicine, anesthesiology, neonatal, pediatrics, surgery cardiovascular surgery) of a 550-bed tertiary hospital in Van, Turkey. This study was approved by the local ethical committee.

### Sample Collection

Following the admission of patients to relevant ICU's, 2 rectal swab samples were taken within the first 24 hours and another one was taken every subsequent 15 days. Rectal swab specimens were taken with sterile ecuvions and transported to our hospitals microbiology laboratory with Amies transport medium (Lp Italiana Spa). All samples were cultured and Enterococci and Klebsiella presumptive colonies underwent identification via the Phoenix automated system (Becton Dickinson, USA). Samples that showed growth were assessed via PCR (GeneXpert®vanA/vanB, Cepheid, USA and GeneXpert®CarbaR, Cepheid, USA) for subtype determination. Samples taken every subsequent 15 days were assessed only with culture.

### Culture and Identification of Samples

Samples that were planned on being cultured were inoculated to Bile Esculin Agar (BEA, Oxoid, England) and Eosin Methylen Blue Agar (EMB, Oxoid, England) before being incubated at 37°C for 24 hours. Presumptive colonies such as black colored colonies that reproduced in BEA and mucoid colonies that reproduced in EMB agar were tested for gram staining and catalase enzyme activity. Enterococci presumed colonies were additionally tested for L-pyrrolidonyl-β-naphthylamide (PYR; Remel, USA) while Klebsiella presumed colonies were additionally tested for citrate and MIL (motility-indole-lysine iron agar) (HIMEDIA, India). Colonies that were indol negative, un-motile and citrate positive and were therefore presumed as *K. pneumoniae* were passaged and pure cultures were obtained. The Phoenix BD machine (Becton, Dickinson and Company, USA) was used in order to identify subtypes and determine antimicrobial susceptibility. Antibiotic susceptibility results were evaluated according to EUCAST (European Committee on Antimicrobial Susceptibility Testing).

### Genotypic Determination of VRE and KPC

GeneXpert®VanA/VanB (Cepheid, USA) and GeneXpert®CarbaR (Cepheid, USA) kits were used for Enterococci and KPC, respectively. This method is a system that detects VanA and VanB in 45 minutes, and K. Pneumoniae genotypic subtypes in 48 minutes via real time automated PCR. This system has a 97.1% sensitivity and 91.2% specificity for VanA, a 82.4% sensitivity and 87.3% specificity for VanB, and a 96.7% sensitivity and 82.3% specificity for VanA/VanB. This systems senstivity and specificity for KPC is 96.6% and 98.6%, respectively. Swab samples for enterococci were initially placed within a buffer solution and vortexed for 10 seconds before the solution was completely transfered to VanA-VanB cartridges. The cartridge barcode was scanned and patient datum were loaded to the device and the start button was pressed in order to begin PCR. Following the preperation of the 0.5 Mac Farland bacteria solution for Klebsiella, a second 1% solution was also prepared. The bacterial suspension was drawn by a pasteur pipette upto the marked line and transferred to a CarbaR cartridge as recommended by the manufacturer. The cartridge barcode was scanned and patient datum were loaded to the device. Then the start button was pressed in order to initiate PCR.

### Statistical analysis

Dependent two group proportion comparison was made for VRE and KPC positivity ratio using the minitab v14 program.

## Results

### Screening test results

A total of 316 rectal swab samples from 230 patients (73 Pediatric, 60 Anesthesiology, 70 Internal Medicine and 27 Neurology ICU patients) were enrolled into the study.

A total of 36 VRE and 7 KPC growths were detected from 316 rectal swab samples. Forty-seven patients were screened at least 2 times. Rest of the patients were not further screened due to discharge, exitus or transfer to other wards within the first 15 days. According to the dependent two group proportion comparison, the high VRE ratio was found to be statistically significant.

### Features of VRE Positivity

36 VRE growths (35 from the rectal swab samples taken at admission and 1 from the control rectal swab samples) were detected.

The ages of VRE positive patients ranged from 0 to 85 (The mean age was 2 for children and 63.4 for adults). While 29 (81%) VRE strains were confirmed as E. faecium; 7 (19%) were confirmed as E. faecalis, E. casseliflavus/gallinorum and E. raffinosus.

The genotypical distribution of 36 VRE positive strains detected via culture according to GeneXpert®VanA/VanB (Cepheid, ABD) were as follows: 28 VanA, 1 VanB and 3 VanA+VanB. Four strains detected via culture were genotypically determined to be both VanA and VanB negative (Table 1).

**Table 1:** 

Subtype	Number of VRE by culture	Number of VanA	Number of Van B	Number of VanA+VanB	Negative
*E. faecium*	29 (80.5%)	24	1	2	2
*E. faecalis*	4 (11%)	1	0	1	2
*E.casseliflavus/* *gallinarum*	2 (5.5%)	2	0	0	0
*E. raffinosus*	1 (0.3%)	1	0	0	0
**TOTAL**	**36**	**28**	**1**	**3**	**4**

Of the 36 VRE positive patients 4 (11%) of them showed VRE negativity during surveillance (negativity in 3 consecutive screenings). A total of 3 (8%) patients showed no negativity despite being surveilled until the end of the study. 13 (36%) patients became exitus and 16 (44%) were discharged while still VRE positive.

All of the 36 VRE positive patients used multiple antibiotics. The most commonly used antibiotics among patients were glycopeptides (27% vancomycin and 8% teicoplanin), penicillins (25%), carbapenems (19% meropenem and 5% imipenem), 3rd generation cephalosporins (14% cefotaksime, 8% ceftriaxone, 3% ceftazidime and 3% cefoperazone-sulbactam), macrolides (5.5% clarithromycin), 5-nitroimidazoles (8% metronidazole), aminogylcosides (5.5% amikacin), oxazolidones (3% linezolid), polymyxins (3% colistin), antifungals (3% caspofungin and 3% fluconazole) and antivirals (6% oseltamivir and 3% ganciclovir). All except 3 VRE postive patients had history of hospitalization.

### Features of KPC Positivity

All KPC growths (7) showed Oxacillinase-48 (OXA-48) positivity with some also showing New Delhi metallo-beta-lactamase (NDM) positivity (Table 2).

**Table 2:** 

No.	Genetic Structure
1	NDM, OXA 48
2	NDM, OXA 48
3	OXA 48
4	OXA 48
5	NDM, OXA 48
6	NDM, OXA 48
7	NDM, OXA 48

All 7 of the KPC positive patients used multiple antibiotics. Distribution of the most commonly used antibiotics among patients was as follows: 2nd and 3rd generation cephalosporins 3, carbapenems 2, ciprofloxacin 2, vancomycin 2 and ampicillin-sulbactam 1. All except 1 KPC positive patient had history of hospitalization. While all KPC positive patients showed continued KPC positivity during the study, 5 (71%) were discharged and 2 (29%) became exitus before the study was concluded.

## Discussion

Surveillance data has indicated that the incidences of MDROs related infections continue to increase worldwide^7^. The most troublesome MDROs are MRSA, VRE and KPC in hospital settings. Therefore, in this study we investigated the frequency and genotypes of VRE and KPC among most vulnarable ICU patients.

The assessment of enterococci, particularly E. faecium, as important agents of MDROs nosocomial infections was definitively established when they acquired resistance to vancomycin. Further, VRE has remarkable abilities to transfer vancomycin resistance to other bacteria (including methicillin-resistant Staphylococcus aureus). Therefore, it is important to recognize colonization of VRE in patients as clinical infection is almost always associated with faecal colonization with this organism^8^. The most common global concern for nosocomial infections and colonization among VRE is E. faecium^8^ as well as in our study. The World Health Organization recently classified vancomycin-resistant Enterococcus faecium (VREfm) as a high-priority pathogen group requiring urgent development of new antibiotics because of the limited treatment options^9^.

In our study, 82.8% of VREfm (24/29) were found to harbor the vanA resistance gene. Similarly, in other studies, from Turkey, the rate of vanA carriage has been determined to be 84–100%^10^–^13^. Although, it has been shown the vanA type resistance is dominant in the US and Europe, whereas vanB tpe resistance is more frequent in Australia and Southeast Asia^8^,^14^, strains carrying vanB or both vanA/vanB have been reported in various countries including Turkey^10^,^11^,^14^–^16^. Our results are in line with those reported by similar studies from our country.

VRE colonization is especially common among ICU patients because of high risk of gut microbiota disruption due to waning mucosal immunity associated with underlying comorbid medical conditions and frequent antibiotic exposures^17^. Our results show consistency with these known risk factors. We found that all VRE positive patients received various antibiotics alone or in combination. Also all those patients had underlying diseases. The most common one was respiratory tract disease (36%). In addition, 92% of our patients had history of hospitalization.

KPC is one of the most prevelant opportunistic gram negative bacteria in the ICU setting has also gained significance over the past years^18^. However the data focusing on intestinal KPC colonization rates are limited in our country likewise others^19^–^21^.

Production of carbapenem-hydrolysing β-lactamases (carbapenamases), which include NDM, KPC, OXA-48, IMP-1 and VIM is the most common mechanism for carbapenem resistance. KPC generally possess KPC, VIM, IMP, NDM and OXA-48 type beta-lactamase enzymes20. Infections caused by strains capable of producing these enzymes are challenging to treat due to their resistance to multiple drugs^20^. The frequency of these enzymes shows broad geographic distribution and varies by the features of the medical center the study was conducted in. The largest reservoir for NDM-1-producing bacteria is the Indian subcontinent, followed by the Balkans regions and the Middle East which seem to be a secondary resorvoir for these bacteria^23^. KPC enzyme is most common in Greece, Israel and USA. VIM-1 was first isolated from an E. coli strain in Greece before being passed on to K. Pneumoniae and spreading rapidly across Europe to eventually cause an endemy. OXA-48 was first revealed in Turkey before being transmitted across Central Asia and Europe and is the predominant type of carbapenamase in the region^24^.

A study by Chanola et al. in 2017 showed a VDM-1 predominancy among Klebsiella isolates^25^. Similar to this, Jung J et al. showed 66% NDM-1 and only 3% OXA-48^26^. Co-harboring of NDM-1+OXA-48 has been demonstrated in numerous studies and poses important challenges to clinicians due to limited treatment options and high dissemination rates 22,27. As previous studies have defined, the most common type of carbapenamase in our country is OXA-48. A study by Azap et al. detected OXA-48 enzyme in all 16 K. Pneumoniae strains isolated^28^. Another study demonstrated that OXA-48 and NDM-1 carbapenemases in 93 (81%) and 22 (19%) of the total 115 isolates ecaluated, respectively^29^. A similar study by Alp et al. detected OXA-48, NDM-1 and OXA-48+NDM-1 in KPC strains with rates of 91.5%, 4.3% and 1%, respectively^30^. Zarakolu et al. analyzed 279 KPC strains and detected 270 OXA-48 and 1 IMP carbapenamase while 8 strains showed no enzyme activity at all21. Our study also showed OXA-48 positivity among all KPC isolates obtained from rectal swab samples. Our finding of OXA-48 predominancy is consistent with previous studies' results reported from Turkey.

Risk factors for KPC colonization have been defined in many previous studies. Prolonged duration of multiple antimicrobial treatment and hospitalization, comorbidity, old age, history of surgical procedures and inadequate sanitation have been emphasised as significant risk factors^20^,^22^. In our study all patients except one were transferred to relevant ICU's from wards, other ICU's or other medical centers. In addition, all patients used multiple antibiotics and had underlying dieases and histories of surgical procedures. These findings coincide with risk factors ascertained from similar studies.

Finally, this study has several limitations to be considered. First, this study was performed on a relatively small scale in the ICU of a single medical center. Findings may therefore not be transferable to other institutions and larger-scale multicenter studies are required in the future. However, the practices and findings described here might be helpful for infection-control management in other institutions. Second, the evaluation of transmission dynamics in the observation period is limited after admission addressed. However, the screening procedure was sensitive enough to detect MDROs.

## Conclusions

In regard to these results, it can be concluded that VRE and KPC colonization rates upon admission and during ICU stay continue to be among the major concerns for clinicians. Our study is in accordance with other findings and supports the importance of identifying and managing risk factors involved in colonization of the human patients with potentially MDROs during hospitalization, especially in ICUs
